# Incense Stick: An Overlooked Source of Health Hazard

**DOI:** 10.31729/jnma.5286

**Published:** 2020-10-31

**Authors:** Oshan Shrestha

**Affiliations:** 1 Nepalese Army Institute of Health Sciences, Sanobharyang, Kathmandu, Nepal

**Keywords:** *hazard*, *incense*, *Nepal*

## Abstract

Nepal, predominantly inhabited by people driven by the principles of Hinduism and Buddhism, has seen the practice of burning incense sticks for a very long time. There has been an extensive practice of burning incense sticks in temples, monasteries, and even regularly in indoor household settings. This article puts light on the constituents of smoke coming from a burning incense stick and on the possible risks, they possess for occupational hazards and indoor air pollution.

## INTRODUCTION

Incense sticks have thrived well. Its use has become extensive as religious beliefs and its sweet aroma back it up. One doesn't need a citation to believe that the use of incense stick is so overwhelming in temples, especially on weekends and festivals, that the air remains polluted the whole period. People worship with incenses daily at home. However, the evaluation of the number of households having a separate room for worshipping is a matter of further study to assess indoor air pollution.

A typical composition of incense stick consists of herbal and wood powder, fragrance material, staining matter, adhesive powder, and bamboo stick. Upon combustion of an incense stick, smoke from incense burning contains particulate matter (PM), gas products and volatile organic compounds (VOCs).^1^ Particulate matter from incense burning is 45 mg/g on average. It is more compared to that of a cigarette, which is 10 mg/g.^2^

In a study of particulate and carbon monoxide (CO) associated with burning incense and cigarette, it was concluded that incense generated more persistent aerosols, greater particulate mass, and a greater ratio of particulate mass to CO mass concentration. A study carried out using Salmonella typhimunium concluded that incense has mutagenic activity, but is weak when compared to that of other tested mutagens.^2^

## MAJOR AIR POLLUTANTS EMITTED

1. Particulate matter (PM): Particles less than 2.5 micrometer (μm) in diameter are referred to as fine particles and are believed to pose the largest health risks because they can go as deep as the alveoli. The combustion of incense, wood, cigarette, and candles are the major sources of residential indoor particulate matter, especially in the 2.5 μm size range and below.^1^ Exposure to particulate matter can aggravate chronic respiratory and cardiovascular diseases, alter host defenses, damage lung tissue, lead to premature death, and possibly contribute to cancer.^3^
2. Carbon monoxide (CO): CO comes from the incomplete combustion of organic compounds. It has a very high affinity towards hemoglobin compared to oxygen. At low doses, it causes headaches, dizziness, weakness, and nausea. At a higher dose, it can be fatal.^1,3^
3. Dioxides: Sulphur dioxide and Nitrogen dioxide can aggravate the pre-existing cardiovascular disease, lung irritation, respiratory illness and alter lung defense system.^3^
4. Volatile organic compounds (VOCs): Volatile organic compounds are chemicals having very low boiling points such that they evaporate easily at room temperature. Common VOCs include benzene, toluene, xylenes, and isoprene. Acute symptoms of VOC exposure include eye/nose irritation, throat irritation, headaches, nausea/vomiting, dizziness, and exacerbation of asthma. Chronic symptoms of VOC exposure include cancer, liver pathology, kidney pathology, and central nervous system involvement.^1^
5. Aldehydes: Aldehydes are another example of volatile organic compounds, which are typically characterized by their irritating properties. Also, they affect nasal mucous membranes and oral passages, producing a burning sensation, bronchial constriction and interfere with mucociliary clearance.^1^ Exposures to formaldehyde are of concern because formaldehyde is a potent sensory irritant and is classified as a probable human carcinogen.^4^
6. Polycyclic aromatic hydrocarbons (PAHs): Acenapthylene, naphthalene, acenapthene, fluoranthene, and phenanthrene are some examples of PAHs.^1^ These compounds have some association with an increased risk of peripheral arterial diseases.^5^

## EFFECTS OF INCENSE SMOKE ON HEALTH

Pollutants emitted from burning incense are said to affect in the same way as passive smoking does. Direct study of the effect of incense smoke pollutants on health is relatively difficult, even though several epidemiological studies have suggested that they do cause health problems.

1. Airway dysfunction: Incense smoke when inhaled causes respiratory dysfunction. Sturton et al in 1996 reported that the incidence of nasopharyngeal carcinoma in Hong Kong in male patients who burnt incense was high as compared with the other malignant cases that were used as controls. They suggested that there is a possibility of incense smoke being the factor in the etiology of this malignant disease.^6^ Also, Ho et al in 2005 investigated the prevalence of chronic respiratory symptoms and acute irritative symptoms among temple workers (as exposure groups) and church workers (as the control group) in Kaohsiung, Taiwan. Their study concluded that working in a temple increases the risk for the development of acute irritative respiratory symptoms, including nose and throat irritation.^7^
2. Dermatological effects: Incense smoke has an association with dermatological problems. Hayakawa et al in 1987 reported a 63-year-old patient, who had practiced incense burning for 15 years. He was found to have itchy depigmented macules on his dorsum manus, left shoulder, and abdomen. A 48-hour closed path test revealed that the perfume in the incense was its cause. It was suggested that the perfume and airborne particles coming from the burning incense contacted the skin and caused the allergic contact dermatitis accompanied by depigmentation.^8^
3. Neoplasm: A study carried out using Salmonella typhimunium concluded that incense has mutagenic activity, but is weak when compared to that of other tested mutagens.^2^ Lowengart et al in 1987 investigated a group of children in Los Angeles County. The parents of acute leukemia cases and their matched controls were interviewed regarding specific occupational and home exposures along with other potential risk factors associated with leukemia. Analysis of 123 matched pairs showed that there was an increased risk of leukemia for children whose parents burned incense at home and the risk was greater for more frequent users. (P =0.07, OR = 2.7).^9^ As for lung cancer, Koo et al in 1996 conducted four epidemiological studies in Hong Kong for over 15 years to help elucidate the role of ingested and inhaled substances in the etiology of lung cancer. Chinese female in Hongkong have high incidence of lung cancer and only a third of this can be attributed to active smoking. As incense was identified as primary source of nitrogen dioxide and airborne carcinogens, its effect was studied and the study found that it had no effect on lung cancer risk among non-smokers.

## WAY FORWARD

Incense smoke, without any shadow of a doubt, emits various chemical compounds that are potent etiological factors for causing air pollution, airway disease, and other health problems. Long-term exposure seems to put the incense users under more risk of developing a serious health condition. It is necessary for a religious country like Nepal, to carry out studies on the risk of an occupational hazard for temple workers and for the people who are exposed to incense smoke regularly. It is also necessary to study the association between incense smoke and various airway disease and malignancies in non-smokers.

## Conflict of Interest

**None.**
